# Ball Mill Refining in Confectionery Manufacturing: Processing Parameters, Fat Structure, Emulsification, and Rheological Properties—A Review

**DOI:** 10.3390/foods15213827

**Published:** 2026-10-26

**Authors:** Marko Petković, Biljana Lončar, Nemanja Miletić

**Affiliations:** 1 Faculty of Agronomy in Čačak, University of Kragujevac, Cara Dušana 34, 32102 Čačak, Serbia; n.m.miletic@kg.ac.rs; 2 Faculty of Technology Novi Sad, University of Novi Sad, Bulevar Cara Lazara 1, 21000 Novi Sad, Serbia; cbiljana@uns.ac.rs

**Keywords:** ball mill refiner, chocolate, compound, particle-size distribution, rheology, emulsifier, cocoa butter, crystallization, energy consumption, conching

## Abstract

Ball mill refiners (BMRs) are widely used in confectionary manufacturing, particularly for producing chocolate, compound coatings, and nut-based pastes by integrating size reduction, dispersion, and partial conching into a single compact unit. This review critically examines the effects of key operational parameters—such as agitator shaft speed (ASS), refining time (RT), grinding media mass (GM) and thermal control—and their cascading effects on energy consumption (EC), particle size distribution (PSD), rheological properties, and final product quality. Recent literature was synthesized to examine how specific mechanical variables interact with different fat systems, including cocoa butter and alternative fats, and emulsifiers such as lecithin and polyglycerol polyricinoleate (PGPR), thereby influencing crystallization behavior, texture, and sensory quality. The reviewed findings indicate that greater refining intensity does not necessarily result in improved product quality, since excessive particle size reduction increases the specific surface area and may increase viscosity and fat requirements. The effects of processing parameters are strongly formulation-dependent, particularly in systems containing alternative fats and different emulsifier combinations. These relationships demonstrate that optimizing BMR operation requires balancing particle size reduction, energy consumption, rheological properties, and the desired quality attributes rather than maximizing refining intensity. Based on the reviewed literature, an integrated approach linking processing conditions, formulation characteristics, and product quality is proposed as a practical framework for BMR process control. Future research should focus on standardized reporting of operating conditions, in-line monitoring, and the development of predictive models that account for interactions between processing parameters and formulation characteristics.

## 1. Introduction

Chocolate production and related confectionery manufacturing traditionally involve distinct, energy-intensive stages, encompassing ingredient mixing, refining, separate conching, tempering, and final crystallization [1]. Among these, refining is a critical unit operation that acts as a fundamental structure-forming step [2]. It governs the final texture, mouthfeel, rheological behavior, and overall quality of suspension foods by reducing the size of suspended solid particles—such as sugar, cocoa solids, and milk powders—above the commonly reported sensory perception threshold of approximately 25–30 μm [1,3,4]. However, refining is far more than a grinding process; it influences subsequent pumpability, coating performance, mold filling, and crystallization kinetics within a continuous lipid phase [2,3,5].

Traditionally, multi-roll refiners (2-roll, 3-roll and 5-roll) paired with separate batch conches have dominated industrial production [3,4]. In recent decades, vertical and horizontal stirred BMRs have gained widespread industrial adoption as versatile, compact, and cost-effective alternatives [1,3]. Operating by agitating a charge of spherical GM (such as high-chrome steel or zirconia ceramics) via a rotating central shaft, these systems combine particle size reduction, dispersion, and partial conching into a single compact, jacketed unit [2,3,6]. This simultaneous execution reduces capital investment, shortens processing routes, and provides exceptional energy-to-surface-area reduction efficiency, making them especially valuable for compound chocolate production where vegetable fats partially or completely substitute cocoa butter [1,3,7].

Despite their commercial success, optimizing BMR refining remains a complex engineering challenge. Balancing critical operational variables—including ASS, residence/refining time (RT), GM size and loading, GT, and thermal management (typically maintained at 40–45 °C)—directly influences EC, PSD, specific surface area (SSA), and rheological properties such as Casson viscosity and yield stress [1,3,8]. Furthermore, mechanical stress intensities alter particle geometry and surface characteristics, while interactions with diverse fat systems (cocoa butter, lauric and non-lauric alternatives, palm-based fats) and specialized emulsifiers (such as lecithin and polyglycerol polyricinoleate—PGPR) affect flow and crystallization behavior [2,3,9].

The influence of refining conditions on product quality cannot be evaluated independently of the characteristics of the processed food matrix [1,2,9]. Differences in fat composition, solid particle concentration, and emulsifier functionality may modify particle interactions, flow behavior, and the response of the material to mechanical stress [1,5,9]. Identical BMR operating conditions may produce different particle size distributions and rheological properties in conventional chocolate, compound coatings, and other fat-based confectionery systems [2,5]. Although refining is generally performed under conditions that maintain the fat phase in a fluid state, the composition and processing history of this phase may influence subsequent crystallization during cooling and tempering. These relationships indicate that refining performance should be evaluated through the combined effects of mechanical processing and formulation characteristics, rather than through particle size reduction alone.

Previous investigations have provided valuable information on individual aspects of ball mill refining, including the effects of operating parameters on particle size reduction, energy requirements, and rheological behavior [2,3,5]. The available results are often obtained using different equipment configurations, product formulations, and experimental conditions, making direct comparisons difficult. In addition, mechanical processing parameters, fat and emulsifier interactions, and crystallization behavior are frequently considered as separate research topics, although their combined effects ultimately determine the technological and quality characteristics of confectionery products [1,2,9]. A critical assessment of these interconnected factors is therefore needed to identify the limitations of existing knowledge and establish a more comprehensive understanding of BMR performance from a food engineering perspective. Although numerous studies have examined individual aspects of refining by BMR, a comprehensive synthesis of the relationships between operational parameters, energy efficiency, formulation characteristics, and quality attributes is essential for process engineers and food technologists [2,3]. The novelty of the present review lies in its integrated assessment of mechanical refining conditions and formulation-dependent responses, with particular attention paid to the interactions among particle size distribution, rheological behavior, fat composition, emulsifier functionality, and subsequent crystallization. Rather than considering these factors independently, the review examines how their combined effects influence processing efficiency and the structural, physical, and sensory quality of confectionery products. The review first describes the principles and operating parameters of BMRs, followed by an assessment of fat systems and emulsifiers and their effects on refining performance. The relationships between rheological properties, crystallization behavior, and final product quality are subsequently discussed, together with the implications for energy efficiency and industrial process control. Current research limitations and future directions are identified, particularly regarding standardized reporting of operating conditions, in-line monitoring, and improved approaches for process optimization [2,3,10].

## 2. Review Methodology and Supporting Literature Mapping

The Scopus database was used as the sole bibliographic source for both the bibliometric analysis and the identification of publications included in the narrative review. The bibliometric analysis was restricted to peer-reviewed publications published between 2020 and 2026 to provide an overview of recent research trends related to ball milling in food processing, with particular emphasis on confectionery applications. The co-occurrence analysis was performed using VOSviewer software (version 1.6.20), based on terms extracted from the titles and abstracts of the retrieved publications. The bibliometric dataset comprised 612 records. In contrast, the narrative review also included earlier seminal and scientifically relevant studies to provide a broader understanding of ball mill refining mechanisms, processing parameters, rheological behavior, crystallization, and product quality. Accordingly, different temporal inclusion criteria were applied to the bibliometric analysis and narrative synthesis to reflect their respective objectives. Although the use of a single database ensured consistency in bibliographic data collection and subsequent co-occurrence analysis, relevant publications not indexed in Scopus may have been excluded, representing a potential limitation of the literature coverage. The search strategy was based on combinations of the following keywords using Boolean operators (AND/OR): TITLE-ABS-KEY ((“ball mill” OR “ball milling” OR “ball mill refin*” OR “stirred ball mill”) AND (chocolate OR cocoa OR confectionery OR “cocoa spread” OR “chocolate spread”)). Studies were considered eligible when they addressed ball milling or ball mill refining in food-related systems. Particular emphasis was placed on studies dealing with chocolate, cocoa-based products, compound coatings, confectionery fillings, and nut-based pastes, as well as studies investigating processing variables, particle size reduction, rheological behavior, energy consumption, fat and emulsifier interactions, crystallization, texture, and sensory or physicochemical quality. Peer-reviewed original research articles and relevant review articles were considered.

**Figure 1:**
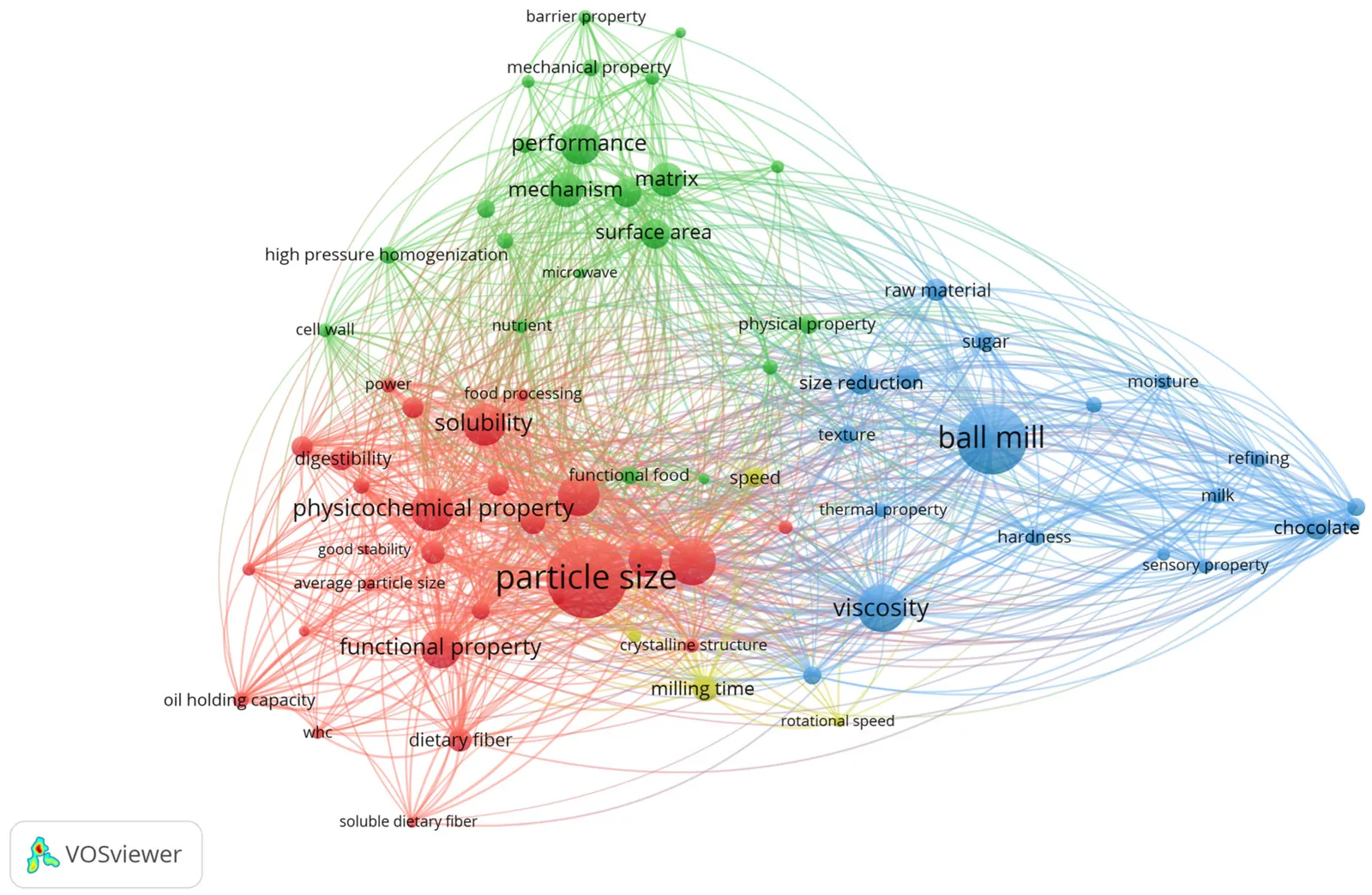
VOSviewer co-occurrence network of terms extracted from the titles and abstracts of Scopus-indexed publications related to ball milling in food processing published between 2020 and 2026 (*n* = 612). Node size represents term occurrence frequency, links indicate co-occurrence relationships, and colors identify clusters of closely related terms.

Studies were excluded when ball milling was applied exclusively to non-food systems, including pharmaceutical, metallurgical, ceramic, geological, or other materials science applications. Food-related studies were also excluded from the core narrative synthesis when ball milling was used solely as a sample preparation step and no relationship with processing performance, particle size modification, rheology, structure, or product quality was evaluated. Conference abstracts, book chapters, patents, editorials, and publications for which sufficient methodological or bibliographic information was unavailable were also excluded. The resulting network visualization is presented in Figure 1. The term co-occurrence network reveals several interconnected research domains related to ball milling in food processing. Particle size occupies a central position in the network, reflecting its fundamental role in linking processing conditions with physicochemical and functional properties. The red cluster is predominantly associated with particle size, physicochemical properties, functional properties, solubility, and dietary fiber, representing the broader use of ball milling for the structural and functional modification of food materials. The green cluster comprises terms related to performance, mechanism, functional properties, and material characteristics, reflecting studies focused on the effects of milling on food matrix functionality. In contrast, the blue cluster is more specifically associated with ball mill, chocolate, refining, viscosity, hardness, and sensory properties, clearly representing confectionery-oriented applications. The close connections between this cluster and particle size emphasize the central role of particle size reduction in determining the rheological, textural, and sensory properties of chocolate and related products. The network highlights the relationships between confectionery applications and the physicochemical, structural, and functional aspects of ball milling represented in the retrieved literature.

## 3. BMR and Processing Parameters

### 3.1. Configuration and Mechanism

**Figure 2:**
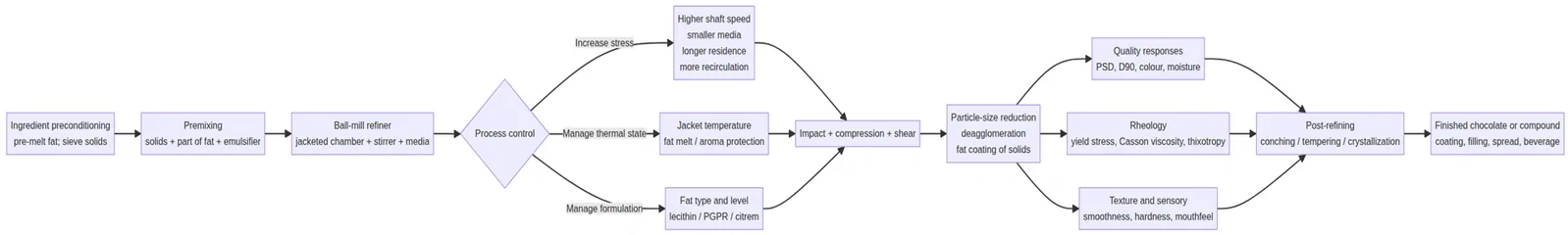
Process pathway and control logic for stirred ball mill refining in chocolate and compound production.

Unlike traditional five-roll refiners that rely on sequential nip-roll compression and high shear, stirred BMRs utilize multi-directional impact and attrition. The primary mechanical components and operational variables govern the stress energy delivered to the food suspension [11]. Figure 2 presents the process pathway and control logic of a BMR. The diagram is an original synthesis based on the mechanisms and variables reported in the cited literature [2,12,13].

A food BMR is generally a vertical or horizontal jacketed vessel equipped with a central shaft and arms or paddles. The chamber is partly filled with GM, commonly stainless steel, steel, or ceramic beads. In chocolate applications, the literature reports that grinding media occupy approximately 60–80% of the available void volume, with bead diameters commonly spanning about 2–15 mm depending on the refining stage and equipment design [2,12]. The feed is a fat-continuous suspension. As the shaft rotates, the media are accelerated and the solids experience impact, compression, sliding, and shear. Particles are broken or deagglomerated at stressing sites, then progressively coated by the lipid phase. Recirculation repeatedly exposes the suspension to the stress field and narrows the coarse tail of the particle size distribution. The process is consequently governed by both breakage and dispersion: a smaller particle size does not necessarily result in improved product quality if the additional surface area is not sufficiently lubricated by fat or emulsifier [13,14].

### 3.2. Important Operating Parameters

It should be emphasized that the effects of BMR operating parameters are strongly dependent on the characteristics of the processed food matrix [15,16,17,18]. Identical refining conditions may induce different structural and physicochemical changes depending on fat composition, solid particle characteristics, moisture content, and emulsifier concentration. For example, increasing agitator shaft speed or extending refining time may promote particle-size reduction and dispersion in conventional chocolate, whereas in reduced-fat or alternative-fat formulations, the same conditions may lead to increased particle interactions, viscosity development, or agglomeration [15,17,18]. Temperature changes may primarily influence fat fluidity during refining, while their effects on subsequent crystallization depend on the composition and thermal behavior of the continuous fat phase [19]. The relationships between processing parameters and product quality should be interpreted in the context of the specific formulation, and optimal operating conditions should not be directly transferred between different food matrices without experimental verification.

#### 3.2.1. Agitator Shaft Speed

ASS represents one of the most critical operational parameters in BMR refining, directly influencing PSR, viscosity development, EC, and product quality attributes. Higher speeds accelerate grinding but increase frictional heat and power draw. Smaller balls provide a higher number of contact points, favoring fine particle reduction, whereas larger balls are required to break down coarse agglomerates. The research indicates that shaft speeds typically range from 40 to 60 rpm in industrial applications, with optimal values depending on specific product requirements and equipment configuration [2,20]:

- PSR. Increasing shaft speed from 40 to 60 rpm results in significant PSR, with D_90_ values decreasing from 53.2 μm to 12.4 μm after 30 min of refining time. The relationship between ASS and PSD follows a non-linear pattern, with sharp reductions observed between 40 and 50 rpm and diminishing returns at higher speeds. This behavior is attributed to increased collision frequency and energy between GM and solid particles at elevated speeds [2,11]. The sensory profile of chocolate and compound coatings is intimately linked to PSD. Particles larger than 35 μm impart a noticeable “gritty” mouthfeel, whereas particles smaller than 15 μm result in an excessive SSA, requiring a larger amount of continuous fat phase to wet the particles;
- Viscosity development. Higher shaft speeds induce increased viscosity in the refined mass, despite smaller particle sizes. This phenomenon results from enhanced structure formation between particles, creating more contact points and interparticle interactions that increase resistance to flow. At 60 rpm and 30 min refining time, Newtonian viscosity reached 1.609 Pa·s compared to 1.186 Pa·s at 40 rpm under identical conditions [2,13,21]. High solids improve particle–particle collision efficiency but exponentially increase slurry viscosity, risking pump cavitation or motor overload;
- EC. Power requirements increase substantially with agitator speed, particularly at values ≥ 80% of maximum capacity. Fišteš et al. [22] demonstrated that EC rises proportionally with shaft speed, with the most pronounced increases occurring when ball mass (BM) exceeds 30 kg at high rotational velocities. This relationship necessitates careful optimization to balance productivity gains against energy costs [22].

#### 3.2.2. Refining Time

RT, typically ranging from 10 to 90 min depending on initial particle size and target fineness, shows linear relationships with most quality parameters. Longer refining times progressively reduce particle size, and D_90_ values decrease linearly across all shaft speed levels [2,12,21], as follows:

- Time-dependent modeling. Recent studies have employed time-dependent modeling approaches to predict water activity, moisture content, and particle size evolution during BMR processing. These models enable real-time process control and endpoint determination, reducing over-processing and energy waste [15];
- Optimal duration. Optimization studies using a central composite design identified 38.5 min at 58 rpm as the optimal condition for chocolate refining, representing a reduction from the previous operating condition of 55 min at 70 rpm. This optimization increased productivity from 109 kg/h to 156 kg/h (43% improvement) while maintaining or improving product quality attributes [20,23].

Rohm et al. (2018) compared roller refining with BMR, and showed that refining intensity affected not only size distribution but also particle shape and surface [13]. This distinction matters because two chocolates with similar D_90_ can have different flow curves if their particles differ in roughness, aspect ratio, porosity or agglomeration state. Process specification should include at least D_10_, D_50_, D_90_ or equivalent span, together with a description of the measurement method. Optimization studies using central composite designs indicate that balancing ASS and refining time can yield significant productivity gains. For instance, optimizing operational parameters in industrial chocolate BMRs has demonstrated capacity increases while maintaining target fineness below 25 μm [24,25]. BMR typically produces particles with a narrower circularity distribution and high sphericity compared to roller milling, because isotropic multi-directional impacts round off sharp crystal edges. However, BMRs often produce a slightly higher fraction of ultra-fine sub-micron particles, which influences the total SSA and rheological profile [26,27].

**Figure 3:**
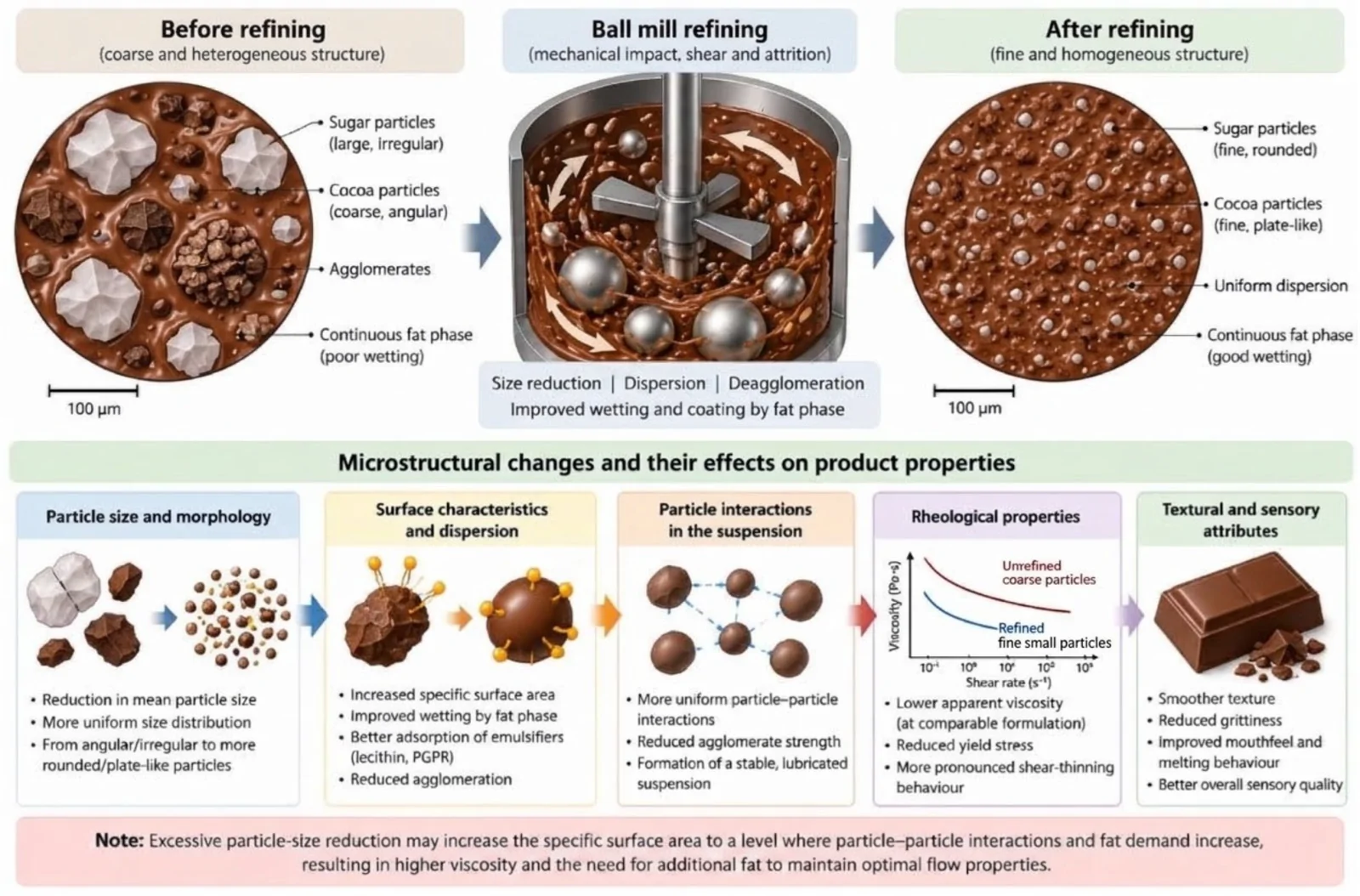
Schematic representation of microstructural changes induced by ball mill refining and their effects on the rheological, textural, and sensory properties of chocolate. Note: AI-assisted tools (Gemini 4 Argon) were used exclusively for the graphical design of this figure. The conceptual framework and scientific content were created and verified by the authors.

The microstructural changes induced by ball mill refining and their implications for the rheological and textural properties of chocolate are schematically illustrated in Figure 3.

Particle size reduction, changes in particle morphology, and deagglomeration modify the specific surface area, particle interactions, and dispersion within the continuous fat phase. These changes influence viscosity, yield stress, and sensory characteristics, particularly smoothness and mouthfeel. However, excessive particle size reduction may increase fat requirements and viscosity due to the greater specific surface area and stronger interparticle interactions. The extent of these effects depends on the formulation and refining conditions.

#### 3.2.3. Temperature Control

Processing temperature strongly affects viscosity, fat crystallization, and overall process efficiency. Optimal temperature ranges of 40–45 °C ensure adequate fat melting for proper mass fluidity while preventing the thermal degradation of heat-sensitive components [2,28], as follows:

- Temperature–viscosity relationship. Viscosity decreases exponentially with increasing temperature, following the Arrhenius equation,
  (1) $$\eta^{50}=\eta^{0}\cdot e^{\frac{E_{a}}{R\;\cdot \;T}},$$
  where *η*^50^ is viscosity at 50 s^−1^ shear rate, η^0^ is the Arrhenius constant, *E_a_* is activation energy (J mol^−1^), *R* is the gas constant (8.3145 J mol^−1^ K), and *T* is absolute temperature (K). *E_a_* decreases with increasing refining time, indicating reduced temperature sensitivity in more refined masses [29];
- Cooling systems. Modern BMRs incorporate sophisticated temperature control systems, including water jackets with electronic monitoring and automatic speed reduction upon increasing product density. These systems prevent overheating from friction-induced heat generation, maintaining consistent product quality throughout the batch [30].

The fat phase must remain sufficiently fluid for transport and wetting, while the product must be protected from excessive temperature that may damage milk-derived components or volatile aroma compounds. Moisture is particularly important in chocolate and compound processing because small amounts of water can increase particle bridging and apparent viscosity [2,12,31]. Recirculation is a second residence-time variable. Higher flow can increase the number of passes and refining rate, but it also increases pump work and may alter the thermal balance. Reporting only batch time is therefore insufficient; flow rate, number of passes or total processed mass should be recorded [32].

#### 3.2.4. Energy Consumption and Efficiency

EC in BMR depends primarily on ASS and BM with secondary influences from product viscosity and throughput. Specific EC for chocolate refining typically ranges from 25 to 45 kWh t^−1^, comparable to other fine grinding applications [7,10,11,33]. The interaction between ASS and BM creates complex power consumption patterns. At low speeds (≤10% maximum), increasing BM from 30 to 40 kg produces a minimal energy increase, whereas at high speeds (≥80%), power requirements become markedly sensitive to BM variations. This behavior reflects the transition from gravity-dominated to centrifugal-dominated ball motion regimes. Process optimization can substantially reduce EC per unit output. The foregoing optimization from 70 rpm/55 min to 58 rpm/38.5 min not only increased productivity by 43%, but also reduced specific EC through shorter processing times and lower rotational speeds [15,34]. Several strategies may contribute to reducing energy consumption during BMR operation. Direct-drive configurations and operation at lower rotational speeds can reduce transmission losses and excessive heat generation, while automated process control may facilitate more consistent operation. From a scale-up perspective, specific energy consumption should be considered together with the required particle size distribution and rheological properties, rather than treating power input as an isolated process variable.

EC in a BMR is not equivalent to motor power alone [17,35]. A useful process balance separates (1) shaft power transmitted to media and product; (2) pumping and recirculation work; (3) jacket heating or cooling and (4) auxiliary energy for premixing, transfer and downstream tempering. Specific milling energy can be expressed as

(2) $$E_{s}=\frac{\int_{0}^{t}P_{\mathrm{mill}}(\tau )\mathrm{d\tau}}{m_{\mathrm{processed}}},$$

where P_mill_ is net mill power, (t) is processing time and m_processed_ is the mass processed.

Higher ASS, GM and recirculation commonly increase stress events, but they also increase power demand and heat generation [36,37]. Longer time may decrease D_90_ while increasing viscosity because of the fine-particle surface area. The optimum is therefore the minimum specific energy that reaches the product quality target, not the minimum particle size. A practical energy efficiency criterion is

(3) $$\eta_{p}=\frac{\mathrm{quality}\;\mathrm{improvement}}{E_{s}},$$

where quality improvement may be defined by a weighted score combining D_90_, yield stress, viscosity, moisture, color, hardness and sensory characteristics. The weights should be selected according to the application.

The management of key operational parameters, such as ASS, GM, and RT, directly affects collision intensity, stress volume, and thermal exposure during BMR refining. To control these effects, operators should continuously track metrics like rpm, torque, fill fraction, and flow rates. Ultimately, avoiding over-refining by stopping at specific quality targets rather than fixed maximum times helps prevent unnecessary increases in viscosity and fat demand.

**Figure 4:**
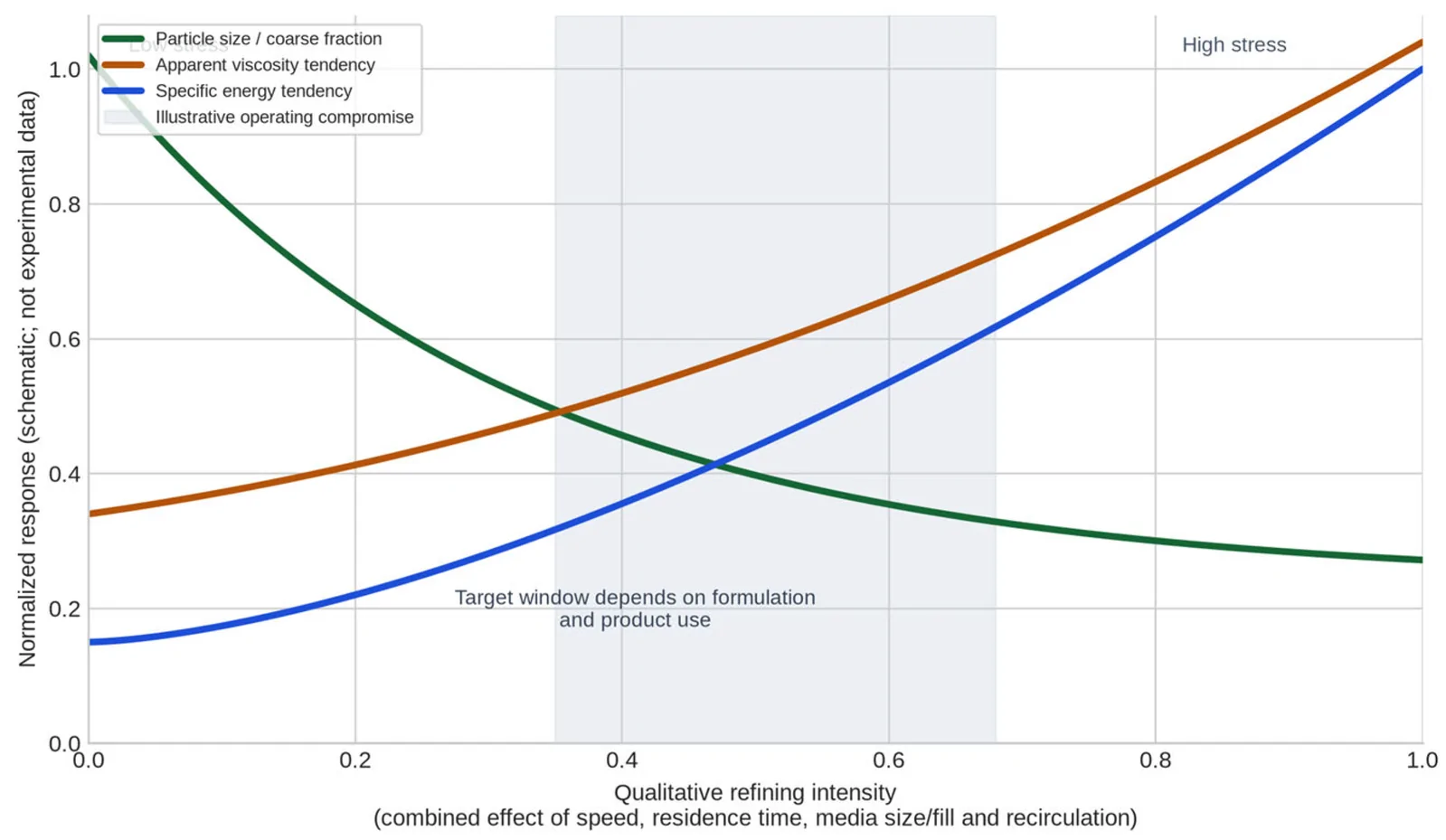
Qualitative parameters (response map for BMR).

The qualitative synthesis of directional responses provides schematic curves rather than experimental data. The shaded zone on these curves highlights that the optimum is ultimately product- and formulation-dependent (Figure 4).

The ranges in Table 1 are literature-reported examples, not universal setpoints. They should not be transferred between mills without matching media geometry, fill fraction, product temperature, solids loading, and target PSD.

## 4. Fats and Emulsifiers

### 4.1. Fats in Chocolate and Compounds

Fat composition strongly influences chocolate rheology, crystallization behavior, and sensory attributes. Traditional chocolate employs cocoa butter as the continuous phase, while compounds utilize vegetable fat blends (typically palm stearin/palm olein combinations) as cost-effective alternatives [38,39].

Cocoa butter’s unique polymorphic crystallization behavior, particularly the desirable form-V (β) crystals, provides chocolate with characteristic snap, mouthfeel, and melting properties. Its triacylglycerol composition, including POP, POS and SOS species, enables several polymorphic forms [40,41,42]. The fat phase is formed after milling, but its crystallization history is influenced by the entire process; ingredient dispersion, shear, temperature, emulsifier composition, cooling rate and tempering. However, cocoa butter’s high cost and supply variability motivate partial or complete substitution in compound products.

The review by Ewens et al. emphasized that reducing or replacing cocoa butter can increase viscosity and hardness and can modify the thermodynamics and kinetics of crystallization [43]. These fats include cocoa butter equivalents, cocoa butter substitutes, lauric fats, palm fractions, coconut oil, oleogels, emulsions, hydrogels and oleofoams. Their performance depends on triacylglycerol composition, solid fat content, melting range, compatibility with cocoa butter and the quantity of non-fat solids. The 2026 review by Balcázar-Zumaeta et al. [38] further emphasizes that molecular packing and long-chain fatty acid alignment determine the formation of crystalline structures, and that controlled tempering is required for an orderly form-V structure [44]. For BMR compound products, this means that fat selection should be screened together with milling, not afterward. A fat that lubricates efficiently at the milling temperature may crystallize too sharply, too softly, or incompatibly during cooling.

Hydrogenated vegetable fats, particularly palm-based blends, offer economic advantages and simplified processing requirements (no tempering necessary). These fats typically comprise 30–33% of compound formulations, providing adequate fluidity during BMR processing while maintaining acceptable texture in finished products. Total fat content significantly influences viscosity and processability [45]. Below approximately 30% fat, chocolate mass loses the fluidity required for efficient circulation through the ball bed, potentially causing processing difficulties. Conversely, higher fat contents (up to 33%) reduce viscosity and facilitate pumping and refining operations. BMRs are increasingly utilized for specialty and cocoa butter alternatives, such as compound coatings based on palm kernel oil (lauric fats), shea butter fractions, coconut oil blends, and plant-based dairy alternatives (e.g., soy milk powder, oat flour and chocolate). Because alternative fats possess different solid fat content profiles and melting dilation curves compared to pure cocoa butter, thermal management during refining by BMR is critical to prevent premature fat seed polymorphic transitions or viscosity spikes caused by heterogeneous fat crystallization [46].

### 4.2. Emulsifier Types and Functions

The successful operation of a BMR relies heavily on formulation chemistry, specifically the type of fat and the inclusion of surface-active agents. Emulsifiers play significant roles in chocolate rheology, reducing viscosity and yield stress while improving flow characteristics for processing (tempering, molding and coating). The two primary emulsifiers in chocolate manufacturing are lecithin (phosphatidylcholine) and PGPR, each exhibiting distinct functional properties [36,47].

Lecithin reduces plastic viscosity effectively at concentrations up to 0.5–0.6%, beyond which additional amounts provide minimal further reduction. However, as concentration increases, lecithin also increases yield stress, potentially complicating flow initiation in processing equipment [9,48]. Lecithin is amphiphilic and can adsorb at hydrophilic sugar surfaces, reducing particle–particle interaction and plastic viscosity.

Lecithin and PGPR do not perform identical functions. PGPR reduces yield stress compared to lecithin, making it particularly valuable for high-viscosity applications. At 0.4% concentration (typical industrial usage), PGPR reduces apparent viscosity by approximately 30%, compared to 40% for lecithin. PGPR-stabilized emulsions display Newtonian flow behavior with small, well-dispersed droplets and enhanced stability against phase separation [49,50]. Specific interactions between PGPR and cocoa butter form new structures that create spaces between sugar particles, reducing particle–particle interactions and consequently lowering the viscosity.

Citrem was reported to be the most effective in reducing hardness and rheological parameters [27,51]. Its response depends on dose, moisture, particle surface, fat composition, temperature and the point of addition. The processing objective is not simply to achieve the lowest viscosity, but the rheological behavior required by process parameters—molding, enrobing, spraying, filling or spreading.

Therefore, increasing lecithin typically lowers plastic viscosity, reducing particle interactions, which improves transport and can lower the required cocoa-butter addition, though excess or late addition may alter texture and yield stress [52,53]. Adding PGPR often reduces yield stress to help with coating and pumping at reduced fat levels, while adding citrem reduces hardness and rheological parameters to keep reduced-fat formulations processable. Additionally, increasing total fat improves lubrication and reduces the risk of milling overload—though it changes caloric density and crystallization behavior—while using a multimodal PSD improves packing density, provided that excessive fine particle generation is avoided. Blends of lecithin and PGPR enable tailored rheological properties unachievable with single emulsifiers. Mixtures containing approximately 30% lecithin and 70% PGPR most efficiently reduce yield stress in both dark and milk chocolate [27,54]. Blends of 50:50 and 75:25 lecithin–PGPR achieved the lowest viscosities for dark and milk chocolate, respectively, at total emulsifier concentrations of 4–6 g/kg.

According to current regulations, the dosage of lecithin is Quantum Satis (QS), while PGPR and citrem have prescribed limits. Chen et al. (2017) showed that minor non-triglyceride lipid components can influence cocoa butter nucleation and polymorphism [55]. Saturated phosphatidylcholine and phosphatidylethanolamine at 0.1% *w*/*w*, followed by rapid cooling to 20 °C without shear, accelerated crystallization, stabilized desirable form-V and improved microstructure, gloss and mechanical strength in the investigated systems. This finding is important for refiner design because emulsifiers and phospholipids can have two distinct roles: controlling flow during milling and modifying crystal formation after milling.

A solid-to-fat mass ratio ranging from 65:35 to 80:20 balances processing dynamics, as high solids promote particle breakage via compression, while lower solid levels improve flowability.

The values outlined in Table 2 serve as examples from the literature rather than absolute, universal standards. Therefore, they should not be applied directly to other mills unless the media geometry, fill fraction, product temperature, solids loading, and target particle size distribution are precisely matched.

## 5. Rheological and Crystallization Properties

### 5.1. Rheological Behavior

Chocolate and compound masses exhibit complex rheological behavior influenced by formulation (fat type/concentration, emulsifier type/concentration, PSD) and processing history (refining, conching, tempering).

While traditional chocolate exhibits non-Newtonian behavior with yield stress and plastic viscosity, compound chocolates with high PGPR concentrations may display Newtonian flow characteristics [56,57]. The transition from non-Newtonian to Newtonian behavior occurs as PGPR concentration increases, reducing yield stress to negligible levels. Chocolate and compound masses behave as non-Newtonian, pseudoplastic (shear-thinning) fluids possessing a distinct yield stress (τ_0_). This behavior is mathematically modeled using the Casson equation [58]:

(4) $$\sqrt{\tau }=\sqrt{\tau_{0,\mathrm{Casson}}}+\sqrt{\eta_{\mathrm{Casson}}\cdot \dot{\gamma }},$$

where τ is the shear stress (Pa), *τ*_0,*Casson*_ Casson yield stress (Pa), *η_Casson_* Casson plastic viscosity (Pa s) and $\dot{\gamma }\;$shear rate (s^−1^).

As refining by BMR duration increases, the SSA increases, exposing more bare solid surfaces that absorb free fat and raise internal friction. Consequently, apparent viscosity and yield stress increase due to this extended processing, resulting in stiffer masses unless emulsifier levels or total fat content are adjusted. Without these modifications, such elevated viscosity can challenge subsequent pumping, enrobing, and shell-molding operations [59].

**Figure 5:**
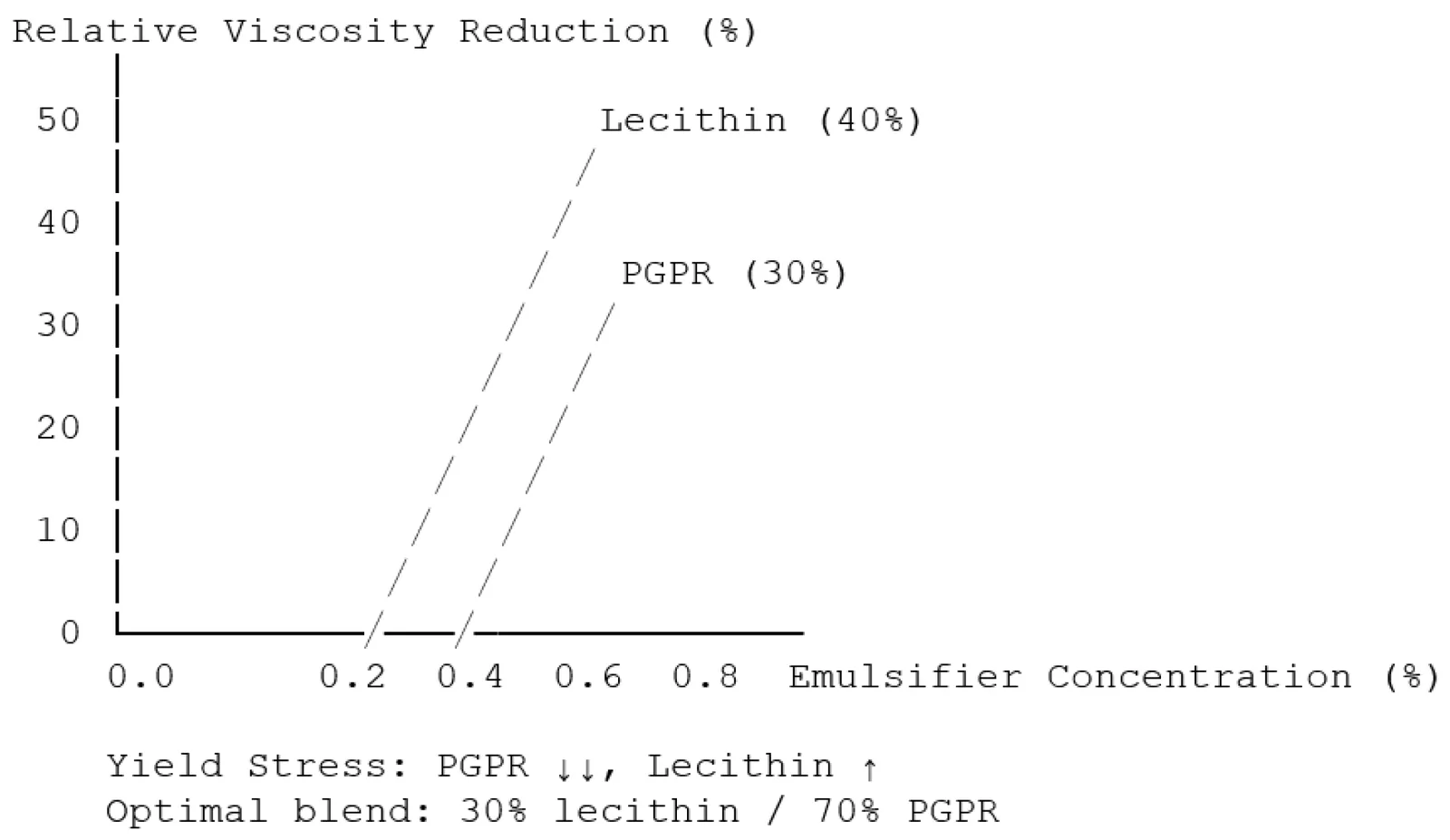
Relative viscosity reduction of chocolate and emulsifier synergy.

The linear trends (Figure 5) illustrate that increasing concentrations of lecithin and PGPR lead to a proportional increase in relative viscosity reduction, with threshold concentrations required before significant changes occur (~0.2% for lecithin and ~0.4% for PGPR). Although individual emulsifiers lower viscosity, their distinct impacts on yield stress—where PGPR significantly decreases yield stress (down arrow) while lecithin may increase it (up arrow)—require optimized multi-component blends. The parameter highlights that a ratio of 30% lecithin/70% PGPR achieves the best functional synergy, balancing plastic viscosity reduction and yield stress control for industrial handling.

**Figure 6:**
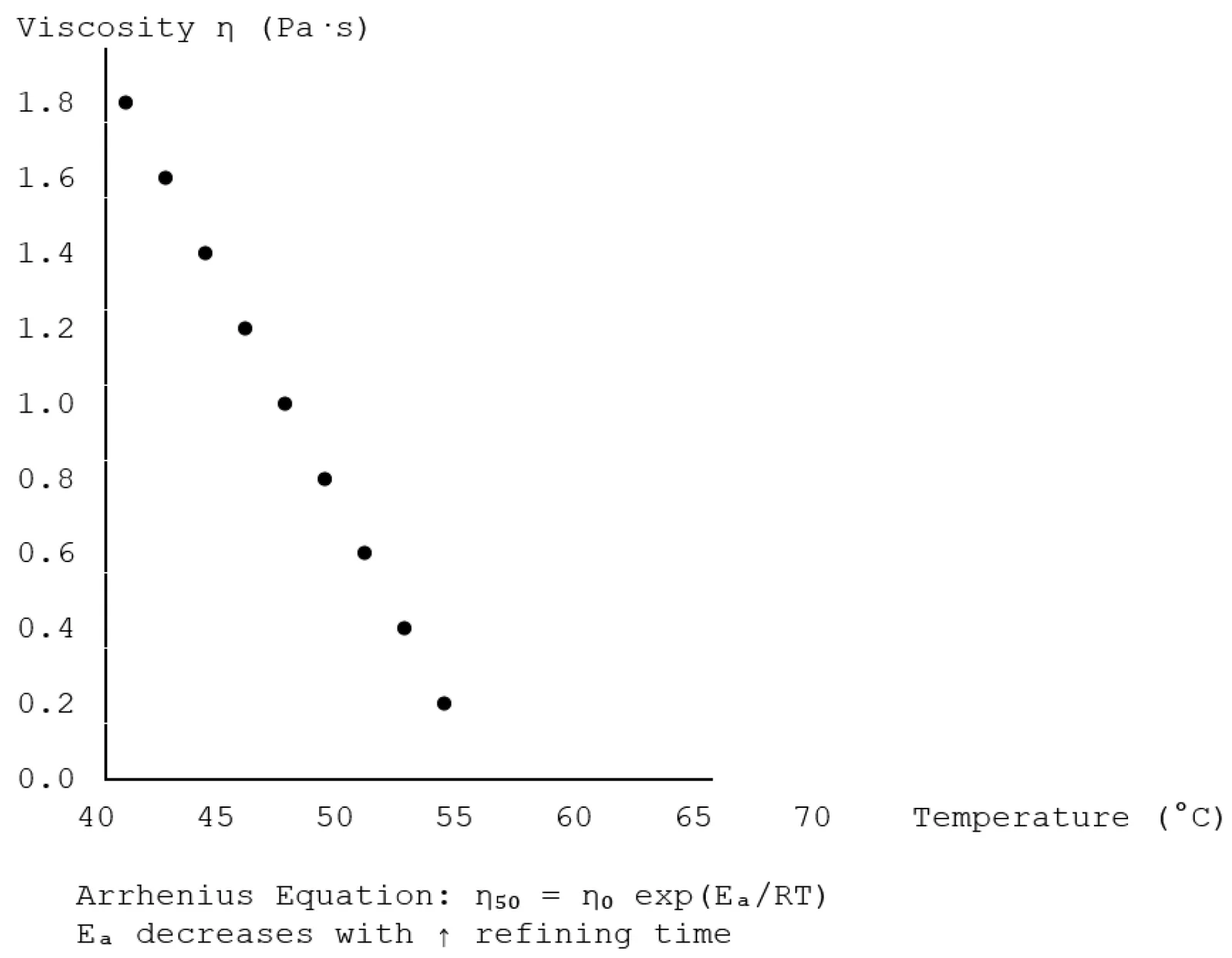
Temperature dependence of chocolate viscosity.

The scatter plot (Figure 6) demonstrates a steep, near-linear decline in apparent viscosity (η) as the temperature increases from 40 °C to 55 °C, reflecting the melting dynamics and reduced internal friction of the continuous fat phase. The note that E_a_ (activation energy) decreases with increased refining time indicates that prolonged mechanical processing alters structural breakdown, reducing the chocolate’s overall sensitivity to temperature changes during processing.

### 5.2. Crystallization Behavior

Crystallization represents an important quality determinant in chocolate and compound manufacturing, affecting texture, appearance, shelf-life, and sensory attributes. Emulsifier type and concentration significantly influence crystallization kinetics and polymorphic form. Increasing lecithin content promotes the formation of β (form-V) polymorphs, the desirable crystal structure for quality chocolate. PGPR accelerates crystallization and seed formation, potentially reducing processing times but increasing susceptibility to over-crystallization [38].

The physical state of the fat matrix following refining by BMR influences the snap, gloss, and contraction properties of the finished confection. Pre-crystallization (tempering) establishes stable form-V crystal nuclei. Research demonstrates that ball-milled chocolate masses containing alternative inclusions (such as plant-based milks) exhibit altered nucleation induction times during controlled cooling [25]. Extended RT promotes a more homogeneous dispersion, leading to faster initial structural organization but requiring the precise adjustment of pre-crystallization temperatures (typically between 28 °C and 30 °C) to prevent fat bloom and ensure proper demolding.

The ratio of lecithin to PGPR affects polymorphic stability, with higher lecithin proportions favoring β (form-V) formation [39,51]. This relationship has practical implications for process optimization, as β crystals provide optimal texture and melting characteristics. Crystallization behavior varies with processing temperature, with PGPR-containing chocolates requiring higher re-heating temperatures than lecithin-only masses to achieve optimum quality. This difference reflects PGPR’s acceleration of seed formation and shorter crystallization times.

## 6. Physical Quality Parameters

### 6.1. Particle Size Distribution

Particle size distribution (PSD) is one of the key quality attributes in chocolate refining because it directly affects mouthfeel, rheological behavior, and sensory perception. In particular, the D90 value is frequently used to characterize the upper fraction of the particle size distribution. Particles above approximately 25–30 μm may contribute to a perceptible gritty mouthfeel, whereas the excessive formation of very fine particles increases the total specific surface area. Consequently, a larger amount of the continuous fat phase may be required to adequately wet the particle surfaces, which can increase viscosity and adversely affect product flow properties [5,50].

Response surface methodology enables the prediction of particle size as a function of ASS and RT, with coefficients of determination (R^2^) exceeding 0.95. For a target particle size of 30 μm, optimal refining times at 40, 50, and 60 rpm were calculated as 28.41, 16.64, and 12.40 min, respectively [2,50]. The relationship between particle size and rheology is non-monotonic in practical formulations. Smaller particles increase total surface area, which can increase the amount of fat immobilized on the particle surfaces and raise viscosity if the fat/emulsifier supply is insufficient.

Conversely, an appropriately broad or bimodal distribution can improve packing; fine particles fill voids between coarse particles, while coarse particles reduce total surface area. Feichtinger et al. [5] found that the particle size ratio, coarse fraction proportion, morphology, and surface properties all affected model chocolate viscosity, and that lecithin reduced viscosity by weakening particle–particle interactions. Toker et al. [2] showed that ASS and RT were significant determinants of particle size, color and steady-state rheology in compound chocolate. Depending on cocoa composition and processing conditions, particle size either increased or decreased, while microstructure, lipid structure, color, and bioactive content changed. This result highlights that dry cocoa powder milling can promote either deagglomeration or aggregation; the outcome depends on fat content, moisture, and energy history [38].

### 6.2. Color Characteristics

Color attributes significantly influence consumer acceptance and perceived quality of chocolate products. The CIELAB color space (L*, lightness; a*, red-green; b*, yellow-blue) provides quantitative color characterization, with derived parameters including chroma (C*) and hue angle (h°) [2,52]. Both ASS and RT increase lightness (L*), chroma (C*), and hue angle (h°) values. This behavior results from reduced particle size increasing SSA and light scattering, making refined chocolates appear lighter. The correlation coefficients between particle size and L*, C*, and h° were −0.9587, −0.7247, and −0.8579, respectively, confirming strong inverse relationships.

Lecithin–PGPR ratios do not significantly alter chocolate color, enabling rheological optimization without compromising visual quality [39].

### 6.3. Texture Attributes

Textural properties, including hardness, fracturability, and snap, are critical quality attributes influenced by formulation and processing.

The incorporation and type of emulsifiers play a defining role in modifying the continuous fat phase and solid–particle interactions. Research demonstrates that increasing lecithin content typically leads to a reduction in both hardness and fracturability [9,44]. This softening effect is closely linked to lecithin’s surface-active properties, which lower viscosity and interfacial tension, thereby modifying fat crystal network formation and hindering tight solid fat packing. Conversely, the addition of PGPR tends to enhance these textural attributes. Because PGPR drastically reduces yield stress and prevents the aggregation of suspended solid particles (such as sugar and cocoa solids), it alters the overall crystallization kinetics and promotes a more compact, rigid internal matrix upon cooling and tempering [53]. These divergent behaviors highlight how specific emulsifiers selectively dictate lipid polymorphism and the strength of the fat crystal network.

Beyond formulation chemistry, physical processing parameters—particularly the refining stage—dictate microstructural attributes that influence texture. Smaller particle sizes generally yield a higher hardness profile [54,55]. This occurs because finer particles provide a greater SSA, resulting in more efficient solid packing and enhanced interparticle friction and contact points within the continuous cocoa butter matrix. However, this relationship has operational limits; excessive refinement can lead to a disproportionately high surface-area-to-free-fat ratio, starving the system of the free fat necessary to lubricate the matrix uniformly. Consequently, over-refining can compromise structural elasticity, leading to a loss of desirable snap and aberrant fracturability.

**Figure 7:**
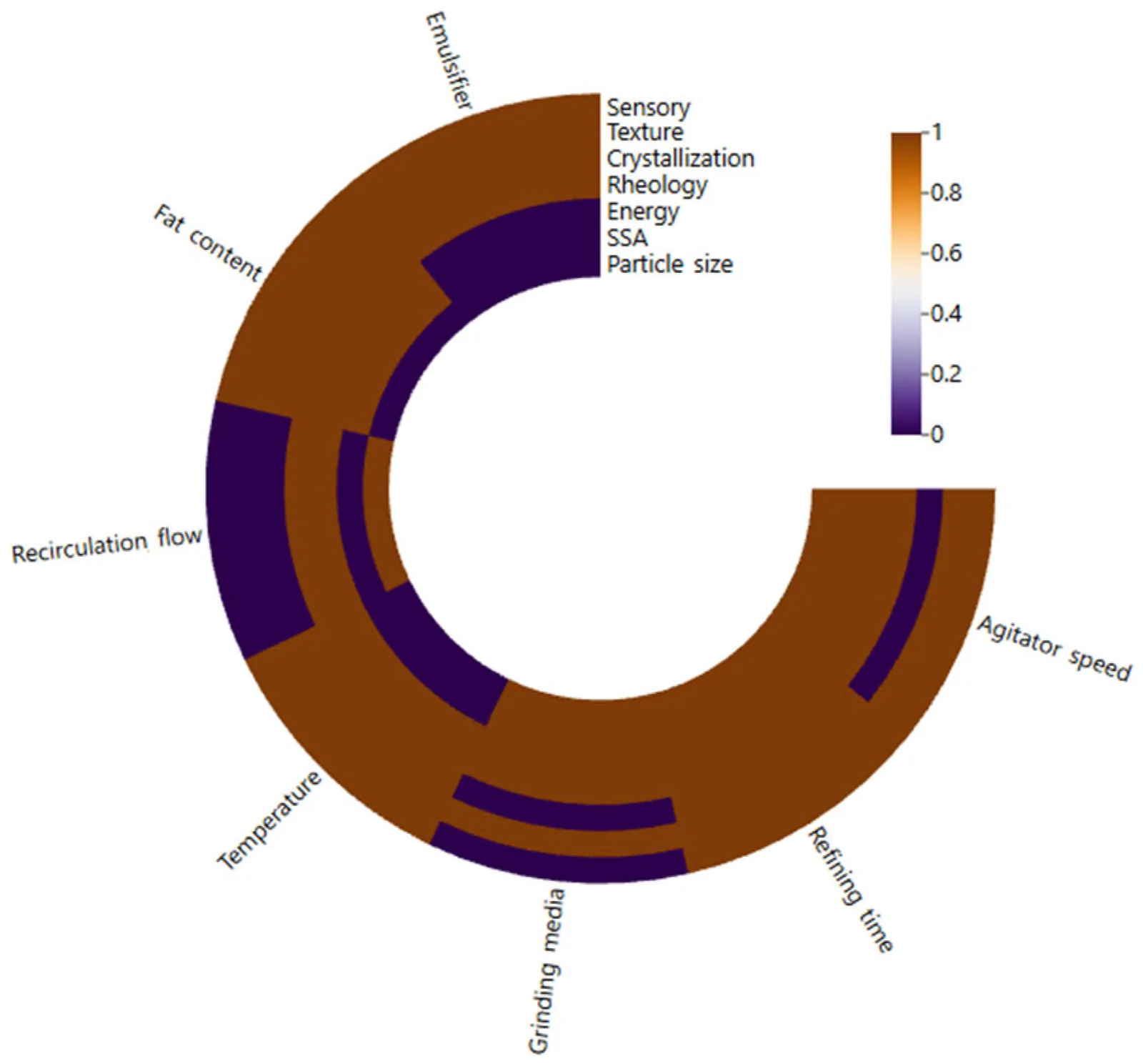
Circular heatmap summarizing the relationships between key BMR processing and formulation variables and selected physicochemical and quality responses identified in the reviewed literature. A value of 1 indicates that the corresponding relationship was identified in the literature considered in this review, whereas 0 indicates that a direct relationship was not identified within the reviewed evidence. The values represent qualitative literature-based associations rather than quantitative effect sizes. The visualization was generated using ChiPlot (https://www.chiplot.online/ accessed on 24 August 2026).

Considering the interdependence of processing conditions, formulation variables, and final product properties discussed throughout this review, a qualitative literature-based synthesis was performed to provide an integrated overview of the principal relationships (Figure 7). The circular heatmap links key BMR processing and formulation variables, including agitator speed, refining time, grinding media, recirculation flow, temperature, fat content, and emulsifier use, with selected physicochemical and quality responses such as particle size, specific surface area (SSA), energy consumption, rheological behavior, crystallization, texture, and sensory properties. The visualization is intended to illustrate the presence of relationships identified across the reviewed literature, rather than their direction or quantitative magnitude.

## 7. Industrial Application, Scale-Up, and Future Research Directions

BMR systems are used across different production scales, ranging from compact laboratory and pilot units for formulation development to industrial batch and continuous systems for chocolate, compound coatings, nut-based pastes, fillings, and spreads. Batch systems, with capacities ranging from approximately 2.5 kg to 20–25 kg per batch provide high flexibility for recipe development and small-lot production, with processing cycles typically lasting 4–8 h. In contrast, continuous industrial systems can achieve throughputs of approximately 500–1500 kg h^−1^ for chocolate and compound products, while low-speed direct-drive configurations can contribute to reduced heat generation and energy consumption. Hybrid processing configurations have also been developed, combining double- or five-roll refining with a finishing BMR to achieve the required final particle size [56,57,58].

Beyond conventional dark, milk, and white chocolate production, BMR systems are applied to vegetable fat-based compounds, hazelnut and filling creams, nut pastes, glazing coatings, cocoa hazelnut spreads, reduced-fat products, and other anhydrous confectionery systems. For some cream and filling applications, a final particle size of approximately 20–22 μm is targeted. Successful industrial operation requires careful control of product and jacket temperatures, generally avoiding temperatures above 45 °C, while adequate fat content is required to maintain mass fluidity and circulation through the grinding media. In the systems considered here, pre-mixed masses containing approximately 30% fat or more provide suitable processability. Nevertheless, formulation-dependent effects remain important, since variations in fat content, particle characteristics, and product composition may promote oil separation in nut-based pastes, agglomeration in cocoa powders, or excessive viscosity in coatings [59].

Despite these industrial applications, the direct transfer of operating conditions between BMR systems remains difficult. Processing performance depends not only on rotational speed and refining time, but also on mill geometry, grinding media diameter and density, filling degree, agitator configuration, product rheology, recirculation conditions, and thermal management. Scale-up should therefore consider specific energy input, stress intensity and frequency, throughput, residence time distribution, and predefined product quality targets, rather than relying solely on rpm and processing time. This becomes particularly relevant for emerging reduced-fat, plant-based, alternative-fat, and nut-based formulations, whose rheological and thermal behaviors may differ substantially from those of conventional chocolate.

The lack of standardized reporting of these parameters further complicates comparisons among studies and limits the transferability of reported optimal processing conditions. Establishing minimum reporting requirements for mill configuration, grinding media characteristics, residence time, and specific energy consumption would therefore improve the reproducibility and practical applicability of the experimental findings [12,13,22].

Several limitations in the available literature consequently remain to be addressed. These include the inconsistent reporting of BMR operating conditions, differences in particle size measurement protocols, limited quantitative information on specific energy consumption, insufficient validation across laboratory, pilot, and industrial scales, and the limited implementation of real-time process monitoring and predictive modeling. Another important research challenge concerns the combined effects of processing conditions and product formulation. Comparative studies conducted under identical refining conditions could help distinguish the effects of mechanical processing from those associated with fat composition, emulsifier type, and solids concentration. This is particularly relevant for reduced-fat and alternative-fat formulations, where changes in the continuous fat phase may substantially influence particle interactions, rheological behavior, and subsequent crystallization [15,17,19]. Future research should therefore move from equipment-specific empirical optimization toward standardized, energy-aware, and quality-driven process control. Particular attention should be directed toward harmonized reporting procedures, the in-line monitoring of particle size, torque, viscosity, and temperature, and data-driven approaches for endpoint determination and process optimization. Energy efficiency should also be considered in relation to the desired product quality, rather than particle size reduction alone [20,22]. Future studies could apply multi-objective optimization approaches to simultaneously evaluate particle size distribution, viscosity, yield stress, specific energy consumption, and refining time. Pareto-based optimization may be particularly useful for identifying alternative operating conditions that balance processing efficiency and product performance. However, experimental validation would be necessary to confirm the practical applicability of the proposed solutions. The integration of mechanistic modeling with data-driven approaches, including artificial neural networks, random forests, and other machine learning methods, represents another promising research direction. Such models could be used to investigate nonlinear relationships between operating parameters, formulation characteristics, energy consumption, and product quality. Their practical implementation requires representative experimental datasets, independent validation, and evaluation across different equipment configurations and processing scales. In the authors’ assessment, the future development of BMR technology should move beyond the optimization of individual processing parameters toward an integrated, formulation-dependent approach combining standardized process characterization, multi-objective optimization, and real-time monitoring. Such an approach could improve the transferability of experimental findings, while supporting the more efficient and consistent industrial production of chocolate, compound coatings, and related confectionery products.

The major research gaps identified from the reviewed literature and the corresponding research priorities are summarized in Table 3.

## 8. Conclusions

Ball mill refining provides an effective alternative to conventional roll refining and separate conching, particularly for chocolate, compound coatings, fillings, and related fat-continuous confectionery systems. The available literature shows that product quality cannot be attributed to a single processing variable. Agitator speed, refining time, grinding media characteristics, temperature, recirculation conditions, and formulation jointly determine particle size distribution, energy consumption, and rheological behavior.

An important practical consideration is that reducing particle size does not necessarily improve overall product quality. Excessive refining increases specific surface area and may increase viscosity and fat demand. Therefore, BMR operation should be optimized against predefined particle size and rheological targets rather than maximum particle size reduction alone. Fat content and emulsifier selection must also be considered together with the mechanical processing conditions because they influence both processability during refining and subsequent crystallization and texture.

Despite the increasing industrial use of BMR systems, comparisons among published studies remain difficult because mill geometry, media loading, specific energy consumption, residence time, and particle size measurement procedures are not consistently reported.

Future studies should therefore focus on standardized reporting, validation across different processing scales, and the in-line monitoring of particle size, torque, viscosity, and temperature. Combining these measurements with predictive and data-driven models may enable more reliable endpoint determination and energy-efficient control of BMR processing.

## Figures and Tables

**Table 1:** Effects of fat composition, solids-to-fat ratio, and emulsifier characteristics on the rheological and processing prop-erties of chocolate and compound products.

Variable	Mechanistic Role	Typical Evidence or Reporting Range	Main Product Consequences
ASS	Changes velocity, collision frequency and shear intensity	40–60 rpm in a compound chocolate study; 450–550 rpm in a planetary cocoa powder study	Usually lowers particle size; can raise viscosity and energy demand when over-applied
RT	Regulates cumulative stress events and residence exposure time during refining	10–30 min in compound chocolate; 10–30 min in cocoa powder; 120–150 min in a compound chocolate study	Reduces coarse particles and moisture; excessive time can increase fine-particle surface area
Ball diameter	Controls stress magnitude and number of media contacts	Approximately 2–15 mm reported for chocolate systems; 5 mm zirconia in cocoa powder work	Larger media increase stress magnitude but can reduce collision number; smaller media favor fine grinding
BM	Determine the number of active grinding bodies and the power draw	Approximately 60–80% of the available void volume in BMR	Higher loading can increase collision frequency but may increase torque and heat
Temperature	Controls fat viscosity, grinding, melting and flavor protection	Jacket control is essential	Too low, poor flow or partial solidification; too high, flavor loss, thermal damage or altered crystallization
Recirculation flow	Increases the number of passes through the grinding zone	Higher flow improved refining rate in chocolate and compound modeling	Lower fineness/coarser surface at comparable elapsed time; higher pumping demand

**Table 2:** Summary of operational parameters, literature ranges, and performance impacts for BMR in chocolate and compound production.

Variable	Mechanistic Role	Typical Evidence or Reporting Range	Main Product Consequences
Solids/fat ratio	Sets suspension concentration, lubrication and available continuous phase 65:35 to 80:20	Strongly formulation-dependent	Lower fat generally increases yield stress and viscosity; higher fat improves flow but changes texture and energy economics
Emulsifier identity and dose	Alters particle–particle and particle–fat interactions	Lecithin, PGPR and citrem are the most relevant recent examples	Changes yield stress, plastic viscosity, thixotropy, hardness and fat requirement

**Table 3:** Current research gaps and future research priorities for ball mill refining in confectionery manufacturing.

Current Limitation	Research Need	Expected Benefit
Inconsistent reporting of BMR operating conditions	Standardized reporting of mill geometry, media size/loading, rpm, temperature and residence time	Better comparison between studies
Different PSD measurement protocols	Harmonized D10/D50/D90 and measurement procedures	Improved reproducibility
Limited real-time monitoring	In-line PSD, torque, viscosity and temperature sensors	Endpoint-based process control
Limited energy data	Reporting specific energy consumption (kWh kg^−1^)	Reliable energy efficiency comparison
Few scale-up studies	Validation from laboratory to pilot/industrial scale	Industrial applicability
Limited studies on novel formulations	Plant-based, reduced-fat and alternative-fat systems	Broader application of BMR
Limited predictive modeling	ML/data-driven process optimization	Predictive control and reduced processing cost

## Data Availability

No new data were created or analyzed in this study. Data sharing is not applicable to this article.

## Abbreviations

The following abbreviations are used in this manuscript:

BMR	Ball mill refiners
ASS	Agitator Shaft Speed
RT	Refining Time
GM	Grinding Media Mass
EC	Energy Consumption
PSD	Particle Size Distribution
PGPR	Polyglycerol Polyricinoleate
PSR	Particle Size Reduction
SSA	Specific Surface Area
BM	Ball Mass
POP	Dipalmitoyl–Oleoyl–Glycerol
POS	Palmitoyl–Oleoyl–Stearoyl–Glycerol
SOS	Distearoyl–Oleoyl–Glycerol
